# A Norm-Creative Method for Co-constructing Personas With Children With Disabilities: Multiphase Design Study

**DOI:** 10.2196/29743

**Published:** 2022-01-06

**Authors:** Britta Teleman, Petra Svedberg, Ingrid Larsson, Caroline Karlsson, Jens M Nygren

**Affiliations:** 1 School of Health and Welfare Halmstad University Halmstad Sweden

**Keywords:** disability, children, norm-critical, participatory design, personas, co-produced care, health care

## Abstract

**Background:**

An increase in the demand for child participation in health care requires tools that enable and empower children to be involved in the co-production of their own care. The development of such tools should involve children, but participatory design and research with children have challenges, in particular, when involving children with disabilities where a low level of participation is the norm. Norm-creative and participatory approaches may bring more effective design solutions for this group. “Personas” is a methodology for increasing user perspectives in design and offers representation when users are absent. However, research on participatory persona generation in this context is limited.

**Objective:**

The objective of this study was to investigate how norm-creative and participatory design approaches can be integrated in a persona generation method to suit children with disabilities in the design of games for health that target this group.

**Methods:**

The method development involved interview transcripts and image-based workshops. Sixteen children with various disabilities participated in persona generation through co-creation of characters and scenarios. The results from the workshops were validated together with 8 children without disabilities, 1 young adult with a disability, and 1 rehabilitation professional. A qualitative thematic design analysis was iterated throughout the process.

**Results:**

The results consisted of an image-based and iterative co-construction method. It was accompanied by examples of personas that were generated and validated within a games for health case. The method showed effectiveness in enabling flexible co-construction and communication. The data resonated with social model perspectives, and the development is discussed in terms of participation levels, salutogenic descriptions of barriers, and norm-creative tradeoffs.

**Conclusions:**

The resulting method may influence future design projects toward more inclusiveness and enable increased representation for children with disabilities in research and design. Using this method to its full potential requires a norm-critical awareness as well as extensive facilitation. Suggestions for further research include the application of the method to design processes in similar contexts or user groups.

## Introduction

### Background

Recent research has shown that while participation is on the agenda of health care professionals, practical guidelines and tools for child participation are lacking [1-5]. This affects children whose lifestyles involve a close relationship with health care services. The pursuit of increased participation for children should also cover research and design processes where children, and particularly children with disabilities, are often left out [6-9]. Child participation is fundamental to ensure a user perspective and to increase the chances for the successful design and implementation of new solutions for children. However, participatory research with children, and especially children with disabilities, has challenges. Since this group has a broad range of special needs, participatory processes depend on customized and user-centered methods [8,10-12]. Overcoming barriers for involvement related to age and disability requires a norm-critical mindset. This allows for a rethinking of power distributions and conceptions of disabilities as barriers. This study thus uses a norm-critical approach to investigate how participatory design methods can enable and capture children’s perspectives to inform design processes that target children with disabilities.

### Co-produced Care: Benefits, Barriers, and Risks

The concept of health care as *co-produced*, as opposed to simply delivered, aims to increase both the quality and efficiency of care [13,14]. Participation is a prerequisite for co-production and has the potential to increase patient empowerment [15,16], motivation, and the effectiveness of interventions [13,17,18]. The benefits of participation for the quality of care have also been found in pediatric contexts. These include better preparation, greater control, feelings of self-esteem, less anxiety, and fewer risks [4,15,19-23]. Research has also shown that children wish to be more involved than they are [24]. Participation is therefore generally regarded as a priority and a prerequisite for good care by health care professionals [4,25]. The *human right* to participate as advocated by United Nations’ Convention on the Rights of the Child [26] thus aligns with this ongoing paradigm shift in health care. Co-produced care channels more control and responsibility over the care processes toward the patient [13,14]. Following this logic, the lesser the patient participates, the greater the risk of lower quality of care. Patients facing barriers for participation thereby risk becoming even more marginalized.

Children with disabilities represent a group that might not manage to fulfil the requirements or norms of co-production unless health care services are designed appropriately. In addition to the patient-professional hierarchy, barriers to co-production are inherent in being a child in an adult domain, such as through parental gatekeeping, communicative inequalities, professionals’ resistance toward power sharing, and a disbelief in children’s capabilities [23]. Despite an awareness of the benefits of child participation, professionals largely fail to achieve this in practice, commonly manifested in failing to address the child [4,23,27]. Moreover, pediatric health care uses a *family-centered approach* in many cases, that has been criticized for blurring the boundaries of who is the real client and risking that the child’s perspective is not prioritized [7,28]. Recent research has thus highlighted the importance of child-centeredness in pediatrics to increase the safety, quality, and perceived value of care [3,5,27-30]. Increasing child-centeredness while at the same time reducing an established family focus must involve additional support for children, since participation requires both involvement and responsibilities. Research-based design methods and tools that empower child patients to independently advocate their own needs and preferences could create such support. One digital category of tools is *games for health* (also called serious games for health [31]). Games for health motivate patients through characteristics borrowed from entertainment games but with health objectives such as maintaining, restoring, and personalizing health [32].

### Involving Children in Design: Participatory Approaches and Norm-Critical Perspectives

The design of tools that aim to increase the participation of children in their own health care needs to be based on the involvement of children from the specific target group. *Participatory design* is increasingly being used to deal with social and health-related issues as it offers a range of creative methods to promote user perspectives [16,33,34]. Participatory approaches might, however, need to be adapted to allow for the inclusion of groups that are too marginalized to get involved through conventional health care fora [10,12,35].

Critical perspectives have come to be influential within this field. Norm-critical and social models of disability are both rooted in critical theory and treat normality, functionality, and disability as malleable and context-bound [36,37]. Norm-criticism aims to identify and question excluding norms within a given context. This involves shifting the focus from a disability-oriented *pathogenic* focus toward a resource-oriented *salutogenic* focus within health care. Salutogenesis acknowledges an individual’s goals, preferences, and resources as keys when working toward better health [38]. Norm-criticism can expose norms and their mechanisms, which in turn can be used as a springboard for *norm-creative* solutions that serve to remodel norms in a direction of empowering practices [37,39,40]. A combined approach based on norm-critical and salutogenic perspectives is thus favorable when seeking to create solutions beyond excluding norms (ie, norm-creative solutions). Unlike explicit norm-critical design, this study thus uses norm criticism as a means and not an end to reach usability and impact. A critical approach is also useful when involving marginalized groups in research and design contexts, in order to challenge power hierarchies and perceptions of barriers [37]. Given that misassumptions about child users are more common than in designs for adults [41,42], this approach relates to the process of identifying and bringing forward children’s preferences and interests in the design process. User participation is necessary to understand user needs [42,43], and a corner-stone for participatory design is to realize that most people can contribute to creative processes when given the right support [11,16,44,45]. Although vulnerable user groups, such as child patients, are becoming increasingly involved in participatory design within games for health [43], such involvement is far from easy.

Challenges in participatory design with children in health care contexts include recruitment (since child patients often have limited energy and spare time) [46], extensive preparations [47,48], and time-consuming data collection (partly since children have more difficulties verbalizing abstract concepts and actions) [27,41,47]. All the above are particularly true for children with disabilities. In addition, when involving persons with intellectual disabilities, there is a need for customized support [49]. Participatory design in games for health projects presents a range of methods and a variation in participation levels, where children sometimes have the role of ideators [50], but more commonly of informants and/or testers [46,51,52]. Importantly, participatory design does not automatically translate into more effective games for health. One meta-analysis showed that involvement in some stages, such as in ideating a game’s esthetics, may even be counterproductive and that children should preferably have roles as informants and co-creators of game challenges [43]. In addition, excessive participation might be energy draining for vulnerable participants and therefore ethically unwise [48]. Having a disability could thereby reduce the possible level of participation, if judged by personal presence. Total participation may thus be both unfeasible and unethical, and entail risks of decreasing the design quality. In order to address these issues, the design method *personas* can be implemented in design projects as a tool for maintaining a user perspective at stages where user engagement is problematic [48,53]. From a child perspective, the personas method offers a longer period of representation throughout the design process than if the child would represent himself/herself in person at only a few stages.

### Personas

*Personas* is a critical user-centered methodology for orienting designers toward user goals by generating qualitative composite archetypes (user profiles) based mainly on qualitative user group data [54,55]. Data-driven personas help designers steer away from assumptions or stereotypes and instead focus on user preferences [56,57]. Furthermore, personas trigger empathy and new ways of thinking [57]. It is thus a goal-driven methodology, which fits with salutogenic approaches. The methodology originated in the interactive design domain, based on the reasoning that if you design for a specific user (visualized as a persona), you will be more successful in reaching users, than if you target “everyone” [54]. Following the introduction of personas in an increasing number of domains, it has consequently been used in the development of games for health that target children [9,41]. Personas move beyond statistical and demographic profiles as they include more detailed, rich, and engaging descriptions. Quotes, images, and details help shape a personality with an individual approach and life situation. Storytelling elements are common to flesh out personality and context (eg, a short story, a day in the life, a situation connected to a specific context, and a more general biography) [55,56].

In order to become believable characters that can have an impact on design, personas must be based on real data and created with consideration for the intended use [56,58,59]. The method requires merging qualitative (and sometimes quantitative) data from numerous people into convincing semifictional characters. Multiple personas can increase usability in the final design, but to rank and limit the number of personas are recommended to keep the design process manageable [55,59]. Personas can be validated through approval from the research team or potential stakeholders [60] and confirmation from participants [59], or through comparison with the data to ensure accurate reflection [55]. Given its flexible user-centered approach, personas as a method lends itself to participatory construction. However, this has not been extensively studied in the context of children with disabilities, as a general method for this is lacking. Against this background and with the described approaches, the personas methodology was seen as a suitable approach for further development through this study.

### Objective

The objective of this study was to investigate how norm-creative and participatory design approaches can be integrated in a persona generation method to suit children with disabilities in the design of games for health. The aims of such a persona generation method would be easier involvement and stronger representation of children with disabilities in design processes where they are the target group.

## Methods

### Setting and Study Design

Ethical approval was granted by the Regional Ethical Review Board at Lund University, Sweden (No: 2017/707).

**Figure 1:**
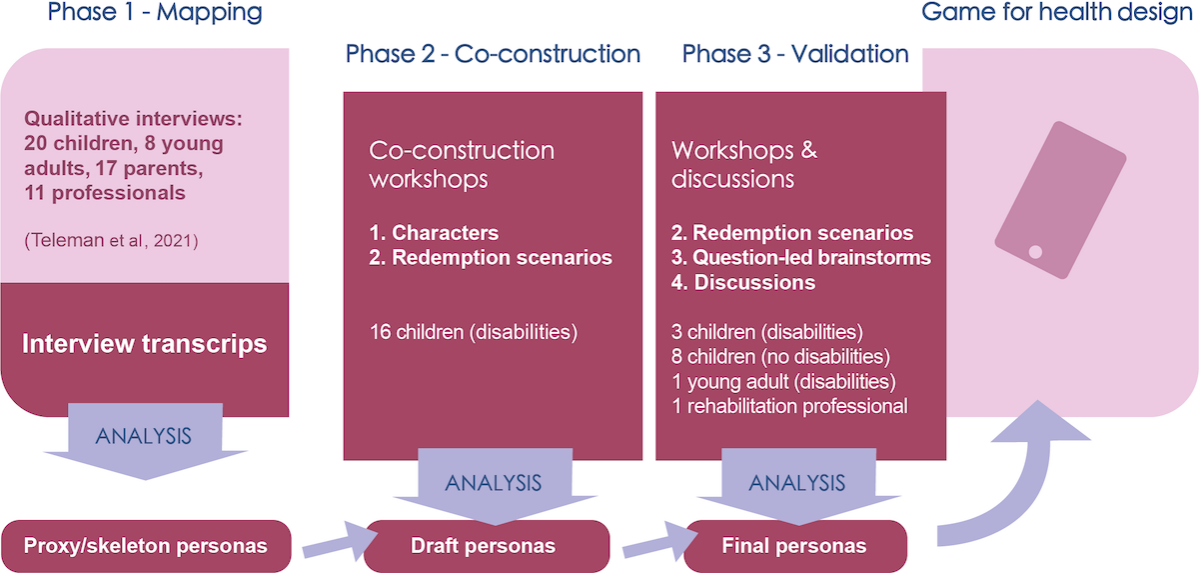
Process overview. The darker parts were included in this study (interview transcript analysis, co-construction, validation, and persona modeling) within the context of a game for health case. The application of the generated personas in the design of the game for health was not covered in this study.

In order to contextualize method development, a game for health design process conducted at a university college was used as an empirical case. The development was thus conducted so that the personas generated through this method could be applicable in the design of such a game (Figure 1). The target group for this game for health was children with disabilities, for whom it was to function as a digital decision support tool (accessed as a tablet/mobile app) to strengthen participation in decisions related to pediatric rehabilitation.

A persona generation method was merged with a participatory design method, inspired by the work of Pruitt & Adlin [55] and Spinuzzi [34]. This merger was further remodeled to include steps where children could co-create characters and scenarios as proposed by Wärnestål et al [48]. The result was a unique study design with 3 main phases (Figure 1). It enabled interpretation and meaning creation from participant input in multiple iterative steps. The emerging data were explored through an inductive thematic design analysis. The 3 phases were as follows:

(1) *Mapping phase.* Data (interview transcripts) from the overarching games for health case were utilized. It involved extracting and analyzing *factoids* (pieces of information) from the data to construct proxy/skeleton personas.

(2) *Co-construction phase.* This phase consisted of creative workshops in which children co-constructed characters and redemption scenarios through images and storytelling. The output was analyzed to draft personas that were continually enriched and then ranked.

(3) *Validation phase*. The draft personas were used in workshops and discussions with the target group and other actors, which contributed to the validation and finalization of the personas.

### Recruitment and Participants

The interview transcripts analyzed in Phase 1 included the following 4 groups: children with disabilities, young adults with disabilities, parents of children with disabilities, and professionals working in pediatric rehabilitation (Figure 1). Only children were recruited for the workshops in Phases 2 and 3. The inclusion criteria were age 6 to 18 years and having an established contact with pediatric rehabilitation services in southern Sweden, thereby having one or more disabilities. The children had to be able to participate in a workshop setting and communicate either orally in Swedish or via any of the augmentative and alternative communication tools used in their rehabilitation. These criteria were based on the resources and skills of the research team. Professionals helped determine which children met the criteria, and these children were invited to participate. The participating children were 6 to 17 years old and had a sociodemographic spread. There were 10 females and 6 males. Various disabilities were represented, including physical, cognitive, and intellectual disabilities, and autism spectrum disorder. Some children had multiple disabilities. Both the recruitment and workshops involved a speech and language therapist experienced in rehabilitation work with children with disabilities [53]. An additional group of children aged 10 to 12 years without disabilities was recruited through a local school for some validation workshops (question-led brainstorming). These workshops aimed to assess the personas’ usability in a design activity, which could involve people outside of the target group (see the Ethics section). The validation phase also involved 1 young adult with a disability (ie, formerly in the target group) and 1 professional working in pediatric rehabilitation.

### Data Collection

An iterative persona modeling process took place during all study phases, informed by the analysis of each phase as shown in the bottom row of Figure 1. Each phase involved different activities, for example, rounds of workshops. A workshop overview is presented in Table 1.

**Table 1:** Workshops performed in Phases 2 and 3.

Workshop	Purpose	Participants	Activity	Output	Persona modeling
1. Characters	Get insights on the target group’s Life situation Personality Interests Frustrations Goals	Children with disabilities	Co-creating collages of visual characters using templates and an image bank	Thirteen child characters	Drafts of 3 portrait-and-bullet point personas merged from characters and skeleton personas Simplified personas
2. Redemption scenarios	Get insights on the target group’s strategies to manage frustrations Test and validate the personas’ credibility and usability for the target group	Children with disabilities	Co-creating strategies in scenarios where personas encounter frustrating situations	Eight redemption scenarios	Persona stories (life situation) Nicknames (personality) Quotes (personality and/or life situation)
3. Question-led brainstorming	Test and validate the personas’ credibility and usability in a design activity	Children without disabilities	Brainstorming on how a game for health could cater for the needs and interests of a persona	Audio recordings, sketches, and notes	Three persona versions for different uses Finalizing personas

Phase 1 (mapping) entailed an analysis of transcripts from 56 semistructured interviews that were part of the ongoing game for health case [5]. The interviews aimed to give an understanding of which experiences and perceptions of participation the children and other stakeholders had. Questions revolved around potential barriers and enabling factors for participation in rehabilitation.

Phase 2 (co-construction) involved 16 children in the roles of both informants and co-creators. This phase consisted of creative workshops. They were either individual, pair, or group workshops (3-5 participants). Each workshop lasted 60 to 120 minutes and was aimed to enable children to contribute with knowledge and creativity through visual input. This input consisted of fictional *characters* in Workshop 1 and of comic strips called *redemption scenarios* in Workshop 2 [48]. The workshops took place in accordance with the participants’ preferences, either at their local rehabilitation center or in their own home, at a time chosen by the family. Children could choose to be accompanied by an adult, although most of the children wanted to participate alone. Well-matched participants were considered important for the group workshops as feeling comfortable is essential for child participation [3,5,61]. Some children knew each other already, which was considered an advantage when forming groups. One workshop included a school class and took place in a classroom during school hours without teachers being present.

Phase 3 (validation) included testing the personas in the construction of redemption scenarios in Workshop 2 (these workshops thus contributed to both co-construction and validation) and in brainstorming related to the game for health case in Workshop 3. The personas were also discussed with 1 young adult and 1 rehabilitation professional at the end of Phase 3. The brainstorm workshops were conducted in a classroom after school hours. There were 2 question-led sessions, with 4 participants in each. The participants were encouraged to write and draw their ideas, and the dialogue was recorded.

### Data Analysis

Both data and method analyses were performed at the end of each workshop round. The analyses were first made individually (by BT and CK). They were then compared, merged, and discussed again in a larger group (including PS and JMN) [62,63]. A qualitative thematic design analysis inspired by Pruitt & Adlin [55] and Kolko [64] was used. Their analysis process is characterized by collaborative (1) visualization and mapping of insights/factoids (eg, by arranging sticky notes), (2) clustering and organizing, (3) finding and visualizing patterns, themes, and needs, and (4) summarizing [55,64]. A similar analysis process was used by Schulz & Fuglerud [53] to create adult personas with disabilities, and by Wärnestål et al [48] when co-creating child personas in vulnerable contexts.

Inputs from all participant groups were extracted from the interview transcripts and turned into factoids in the analysis of Phase 1. The factoids were then abstracted into themes describing user *needs*, which were clustered as notes. Transcripts were made of the visual data in Phases 2 to 3 (ie, output from the workshops). This included listing the images that were used in the co-created characters (and possible comments attached) to find emerging patterns. Visual data from the redemption scenarios were analyzed in a similar manner, where the children’s stories were transcribed into factoids describing user *strategies*. The transcripts generated text or visuals that was mapped to different personas.

### Ethics

Information about the study and the voluntary nature of participation was given to all the participants prior to inclusion. Informed written consent was obtained from parents (this term includes all legal guardians) for the children who chose to participate, as well as from participants over 15 years of age. All personal information was handled according to the General Data Protection Regulation [65] and the Swedish Ethical Review Act. Each child participated in a maximum of 2 participatory activities that were kept short in order not to drain the energy of participants, following the ethical principles of the World Medical Association [66]. While striving for user participation at essential stages, some activities were considered justified to be performed with less vulnerable participants (children without disabilities/young adults with disabilities) for the same reason. Representation in these activities was based on the experience of being a child or having grown up with a disability [47,49].

## Results

### Phase 1–Mapping

#### Interview Transcript Analysis

**Figure 2:**
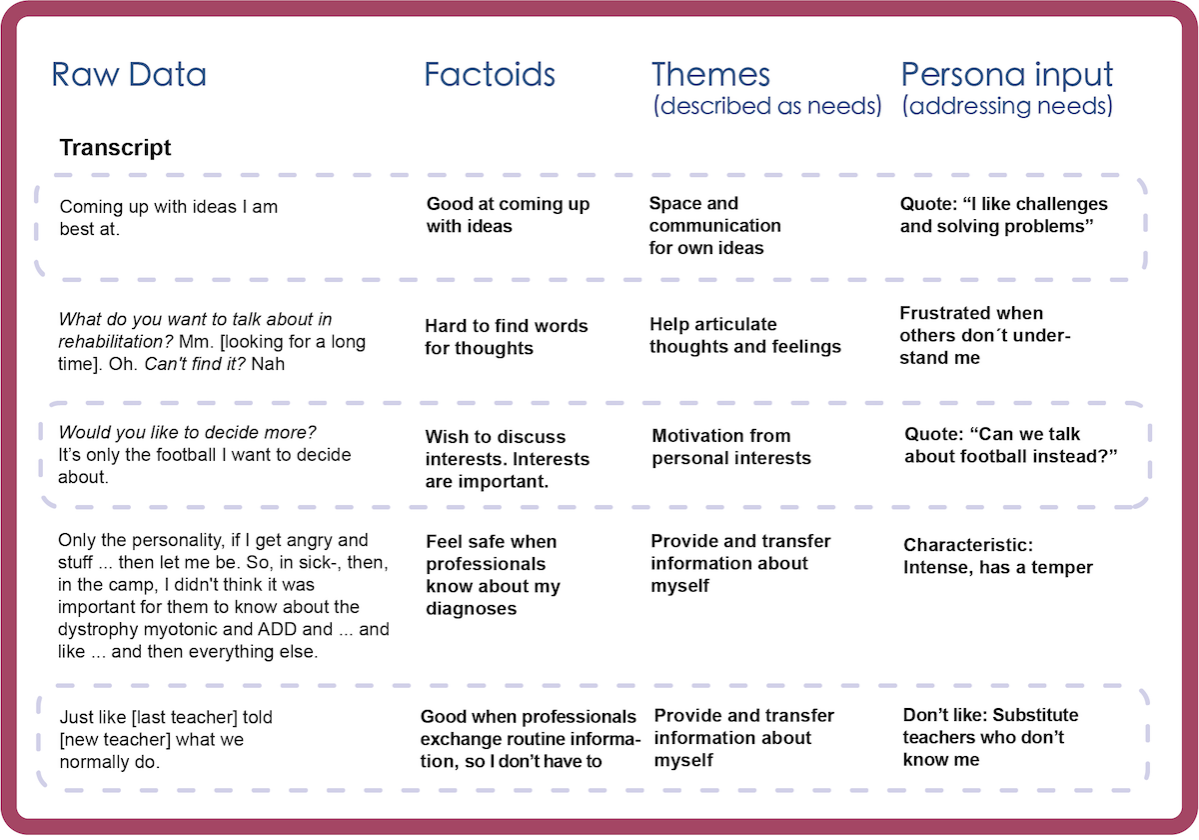
Examples from the data analysis of transcripts from the interviews with children during the mapping phase.

We started to gain an understanding of the participants’ life situations and approaches, and could identify various *needs* in the analysis of interview transcripts. The analysis showed that many of the children were not very talkative unless they had a chance to discuss their own interests, hobbies, or specific issues such as problems with their assistive technology, wheelchair, or similar. Many of them seemed to count on adults to communicate their needs and resources. The interview transcripts also showed that communication through speech was not always easy, and the total data from child interviews were limited in comparison with that from the other participant groups. However, many of the children considered themselves to be creative and good at problem solving. Figure 2 shows examples from the analysis of the interviews with the children.

The interviews with the parents contained rich descriptions of what their children’s daily lives look like. Many expressed concerns regarding their children’s low level of participation and communication struggles. Rehabilitation professionals emphasized the importance of knowing and being “on the child’s level” to be able to communicate. Parents and professionals provided many examples of the children’s needs and how they personally work to accommodate these. Making rehabilitation exercises more playful and planning ahead were examples of this.

#### Modeling: Proxy/Skeleton Personas

A persona construction was initiated based on the mapping phase analysis, and 3 proxy/skeleton child personas were created (Multimedia Appendix 1). Their main purpose was to function as communication tools within the research team and with potential stakeholders in the games for health case. The proxy/skeleton personas helped the team reach consensus about what they should contain and thus which ingredients to search for in order to create the final personas. They provided hints of what kinds of situations, goals, and issues children in the target group are dealing with. These initial versions thus also served as skeletons when developing the final personas.

Both our analysis and proxy/skeleton personas were disproportionately influenced by adults’ perspectives due to the uneven distribution of data. In order to address this, the following phases would have to enable children to express themselves through other means than interviews. Phase 2 therefore consisted of participatory workshops (1 and 2) with the goal of generating inputs that could enrich the proxy/skeleton personas (Table 1).

### Phase 2–Co-construction

#### Workshop Preparations and Image Bank

The purpose of Workshop 1 was for the children to generate fictive characters based on themselves. These characters were later to be merged, possibly with the proxy/skeleton personas, and remodeled into final child personas. A visual overview of the design process and a workshop agenda were presented (Multimedia Appendix 2) to gain trust and provide transparency. The workshop facilitators described the role of the children and emphasized the importance of their contribution as users of pediatric rehabilitation. It was also explained that there were no “solutions” or “right answers” to the activity. The facilitators wore casual colorful clothes and sat down with the children as opposed to standing above them to further mitigate the power imbalances between researchers/adults and participants/children. If there were fewer facilitators than children, they moved regularly between participants. It was preferred to not greatly outnumber the children as this could feel intimidating (maximum 2 facilitators per child).

The co-construction workshops had to offer other ways of participation than oral communication. The material also had to suit children of various ages and with various abilities and literacy levels. Visual and physical media allowed the participants to use nonliterate skills. An image bank with cards and character templates was created, besides the visualizations of the process and the agenda. A test workshop carried out with the research team led to some adjustments. It also generated characters of each team member. These served as presentation tools for facilitators as well as examples when describing the activity (Multimedia Appendix 2).

**Figure 3:**
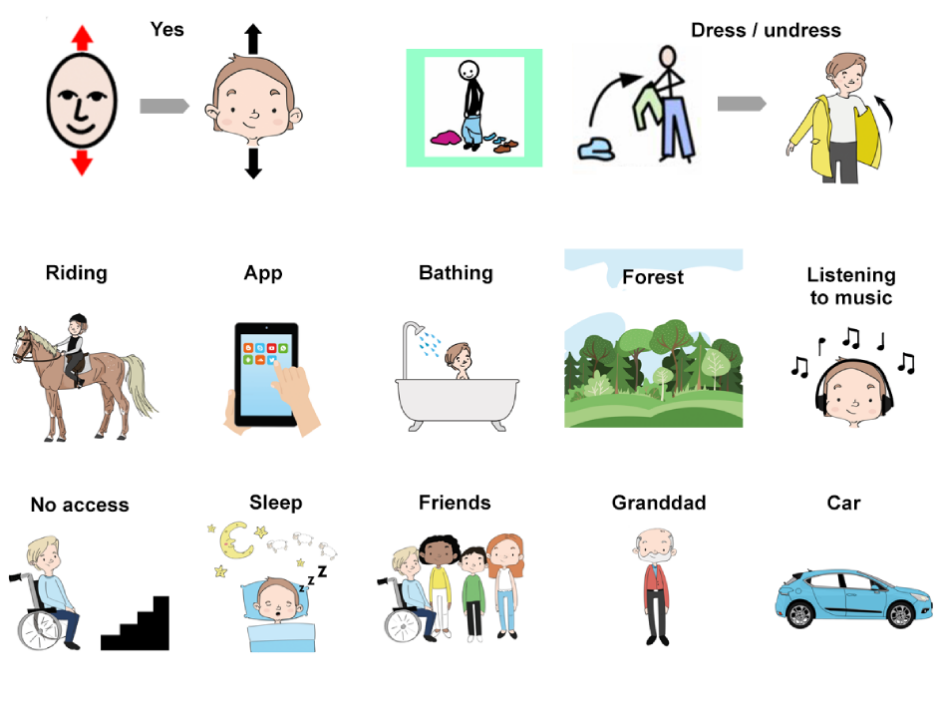
Examples of redesigning existing communication aid images (top row) and new image cards (middle and bottom row).

An image bank of approximately 160 image cards was created with the purpose of enabling and materializing nonverbal and nonliteral input. Semantics and visual references would determine the cards’ ability to motivate and trigger ideas in the participants. We drew inspiration from the most established communication tools used in Swedish rehabilitation to increase clarity and familiarity in the images and kept crucial elements for easy identification. For example, arrows showing movements, a red flash symbolizing pain, symbols for *Yes/No*, and other abstract words were only slightly adjusted to visually match the image bank (Figure 3, top). The redesign of established images was partly a matter of visual coherence and partly about motivation through providing attractive and playful materials. Other images were designed from scratch based on the topics brought forward by children and parents in the interviews, such as loneliness, wheelchair access, or school situations. Finally, we added cards that would help generate foundations for personas, such as different hobbies, moods, or relationships (Figure 3).

The imagery for the workshops was designed to avoid prejudices around, for example, family forms or gender, which followed the norm-critical approach. There were, for example, cards representing a “nuclear family” as well as separate cards for mum, dad, brother, and sister. We also included characters with ambiguous gender and less gender coded outfits. However, the professionals advised us to keep the cards “simple, stereotypical, low in details, and sharp contrast.” In view of the slightly contrasting advice from Wilder [67] that states that children with disabilities are more likely to understand realistic images, we kept cards with animals and objects fairly realistic and less stylized or cartoon-like. It was, however, a question of balance between representation, comprehensibility, and practicality. A limitation on the variety of cards was that we had to keep the number of cards low in order to make the workshop manageable. Each representation of a person could not come in many variations of skin tones for example. The standard face created for the communication cards was slightly cartoonish and androgyne, with brown hair and a relatively fair skin tone (Figure 3, top left). The skin tone generally matched the children who participated in the workshops but could have been altered otherwise. Black and white images were not considered an option, as it would reduce both contrast and appeal. The workshop facilitators encouraged the participants to adjust the cards with the help of pens, glue, and scissors, as we had to use some archetypes where people, things, and places were concerned.

The image bank was to enable and motivate children to elaborate around both tangible and abstract concepts and generate cornerstones for the personas. It included sets with the following themes:

1. A template character, with a choice of accessories, costumes, and items to dress it with and color.
2. Communication aid cards such as *good*, *bad*, *approve of*, *now*, *not*, *thank you*, *who*, *know*, and *boring*. These were scarcely used since participants managed well without them.
3. Moods and personal characteristics such as *smart*, *clever*, *sad*, *crazy*, *nervous*, *curious*, *talkative*, and *fun*.
4. Actions such as *read*, *talk*, *ask*, *sleep*, *remember*, *teach*, *listen*, *look*, *give*, and *hurt*.
5. Chores such as *to clean*, *shower*, *dress*, *do homework*, *brush teeth*, and *go home*.
6. Hobbies and interests such as *bake*, *cycle*, *swim*, *read*, *music*, *sports*, *horse*, and *bird*.
7. Products, aids, and gadgets such as *wheelchair*, *tablet*, *TV*, and *image chart*.
8. People such as *mum*, *dad*, *grandparents*, *friends*, *teacher*, *assistant*, and *health care professional*.
9. Places such as *home*, *school*, *hospital*, *rehabilitation center*, *forest*, and *sea*.
10. Transports such as *car*, *bus*, *airplane*, *boat*, and *taxi*.
11. Struggles such as *crowds*, *no wheelchair access*, *difficulty to focus*, *pain*, and *vision problems*.
12. Blank cards for emerging ideas.

#### Workshop 1: Co-constructing Characters

The activity was briefly introduced and each child started with an A3 template with a blank character in the middle, items to dress it with, and piles of image cards on the side. The image cards were arranged in themes so that the participants could create collages around each theme.

It emerged that it was preferable for 1 adult to sit with each child, not too close to other participants, and guide the child through the process. Although the approach was to ask about the child’s own experience and life situation, a persona is not an actual person, which enabled some fantasy to go into the characters. The participants’ energy levels and attention span influenced the length of the workshops, which lasted between 60 and 120 minutes. This meant that participants considered themselves ready at various stages, which made us discard our initial idea of collectively summing up and reflecting on each character. Notes to remember or explain parts of the characters were added to the collages by the facilitators. The facilitators summarized their observations after each workshop and reflected on what could be improved for the following workshops. This could lead to complements or adjustments to the workshop layout or image bank. Each workshop contributed to a greater understanding of the participants’ life situation and task management.

#### Workshop 1: Output and Analysis

**Figure 4:**
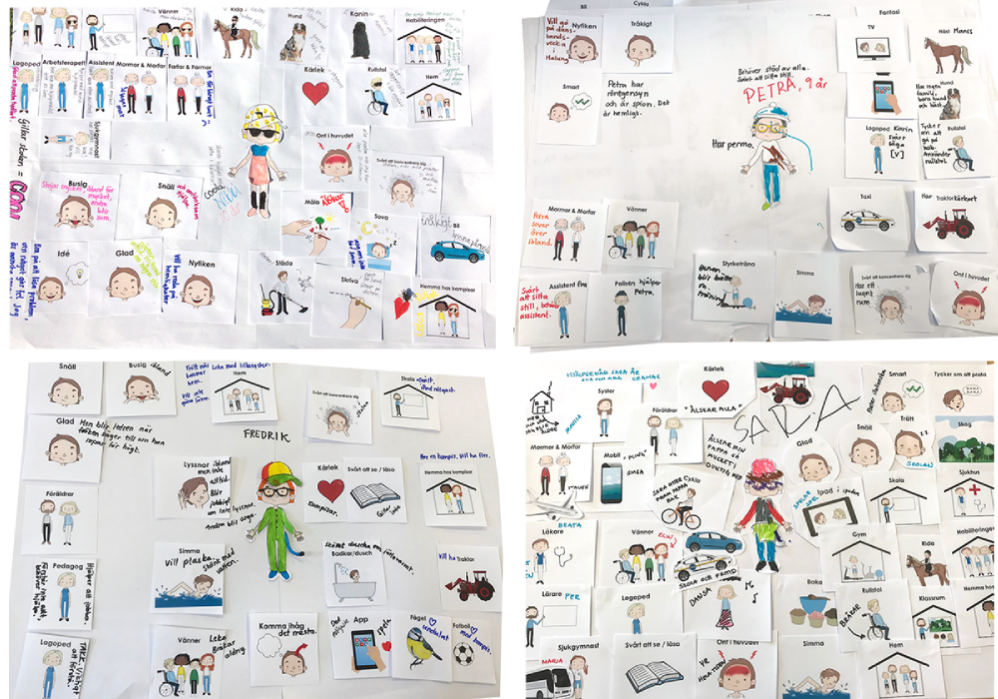
Output from Workshop 1: Co-created characters.

The output from each child (13 in total) was a colorful paper collage of image cards, drawings, and notes that together formed a part biographical part fictional character (Figure 4). The characters were transcribed into lists within the following 7 categories: personality, important relationships, hobbies/interests, frustrations, places, transports, and products, in order to visualize patterns in the data. Each image card’s frequency was also listed to provide insights on common topics within each category. Comments and expressions were also included. Observations of task management could also become persona input, such as whether a persona prefers process control or has a more discovery-oriented approach.

**Figure 5:**
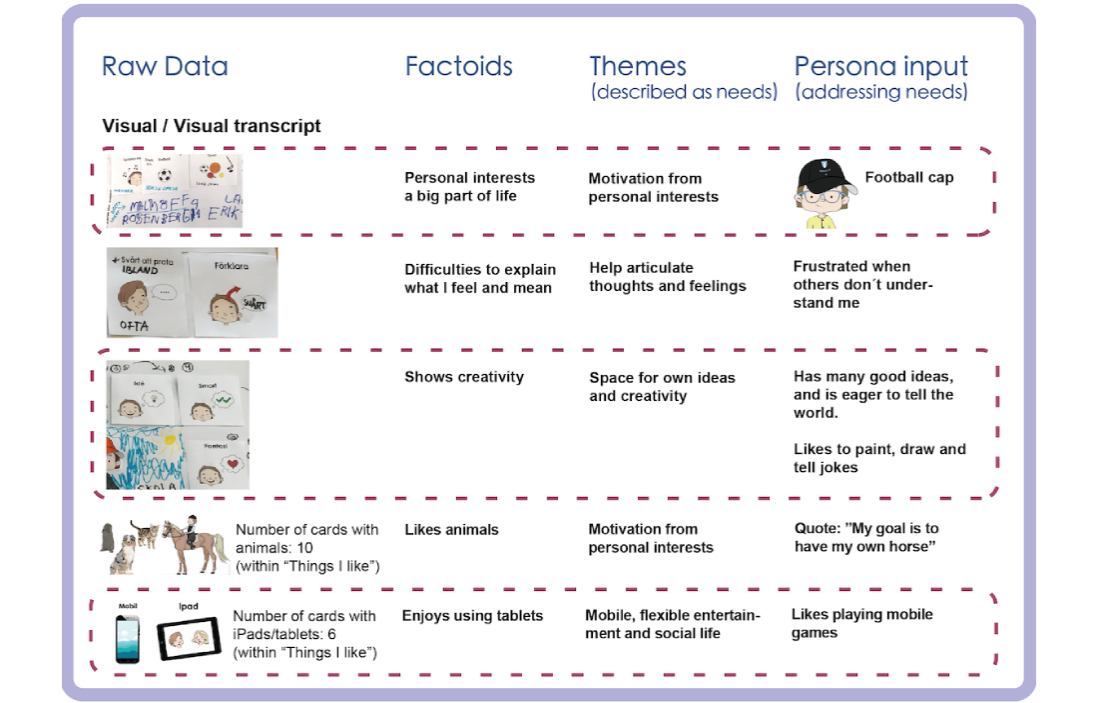
Examples from the data analysis of co-constructed characters in Workshop 1.

The analysis of the characters showed the use of similar cards for describing *personalities* and *frustrations.* Many characters displayed difficulties in expressing themselves or that others had difficulties in understanding them. One example was the statement “my parents need help in understanding me.” Figure 5 shows examples from the analysis of Workshop 1.

Observations of the workshop procedure showed that the image cards and layout had worked well in terms of allowing creativity. The children were imaginative and did not hesitate to adjust the image cards. Some also drew or wrote their own ideas. However, some parts did not suit the participants’ fine motor skills. Small cutout costume pieces (eg, hats) proved difficult to deal with and had to be redesigned. A major part of the facilitation was to help participants stay focused or to move on to the next theme. It became clear that it was more difficult to concentrate in large groups than in individual or pair workshops. This was probably due to both noise and distraction levels, and that the facilitators had to walk around between participants. We reduced group sizes to have 1 facilitator per child further into Phase 2, which provided a calmer environment and more effective assistance. Some participants with a disability from the autism spectrum preferred a more structured process. We arranged the themes in order to address this and made an overview sheet showing all the cards within each theme. We also realized that if the participants became bored or tired, they might consider the workshop as finished. In order to help them distribute their energy evenly during the activity, we had to clarify what was expected to be covered on the A3 paper. Spending too much time creating the character in the middle (which could easily be perceived as the main part) could thereby be avoided.

#### Modeling: Creating Personas

**Figure 6:**
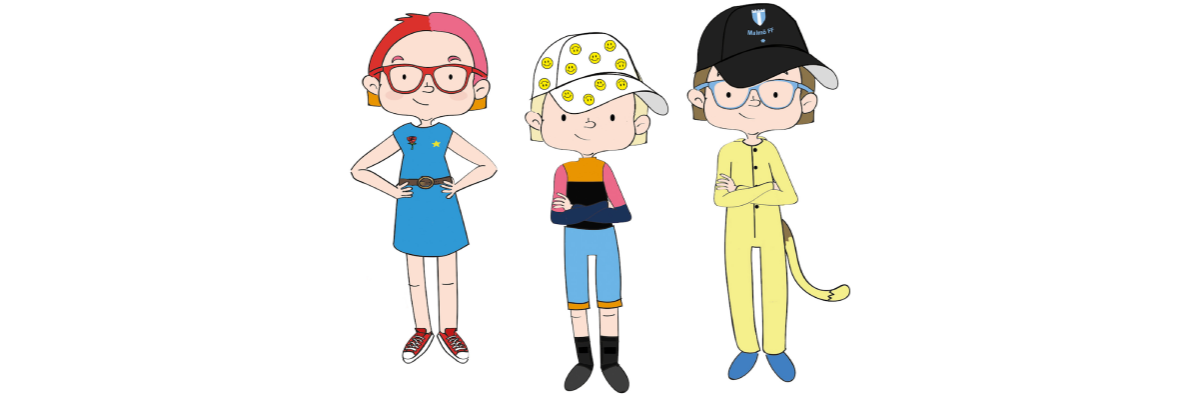
Visual portraits of the personas, modeled from input of Workshop 1.

In order to create personas out of the 13 co-constructed characters, they were divided into 5 groups based on similarities in *personalities* and *frustrations* (not disability). Three personas were considered suitable for this study. Three of the groups were thus selected, representing a variety of the aforementioned aspects. Each group was then merged into 1 persona by combining elements from all its characters. The personas now had a visual portrait and bullet points under the headings: I am good at, Family, Personality, Frustrations, Goals, Motivations, Products, I like, and I don’t like. We prioritized input from children we knew wanted to contribute again when choosing between equivalent alternatives. Bringing forward visual details from the characters could generate sympathy from both recurring and new participants in the upcoming workshops. The proxy/skeleton personas were revisited and were used to flesh out the new personas. Adjustments were made to ensure that the personas expressed coherent characteristics and abilities. Personas were kept androgynous in terms of name and appearance when based on children from different genders. Appearance and clothing were influenced or copied from the children’s characters. Assistive technology or aids were described, but disabilities were not explicit. Figure 6 shows the visual portraits of the 3 personas *Molly*, *Kim*, and *Alex*.

#### Workshop 2: Co-constructing Redemption Scenarios

**Figure 7:**
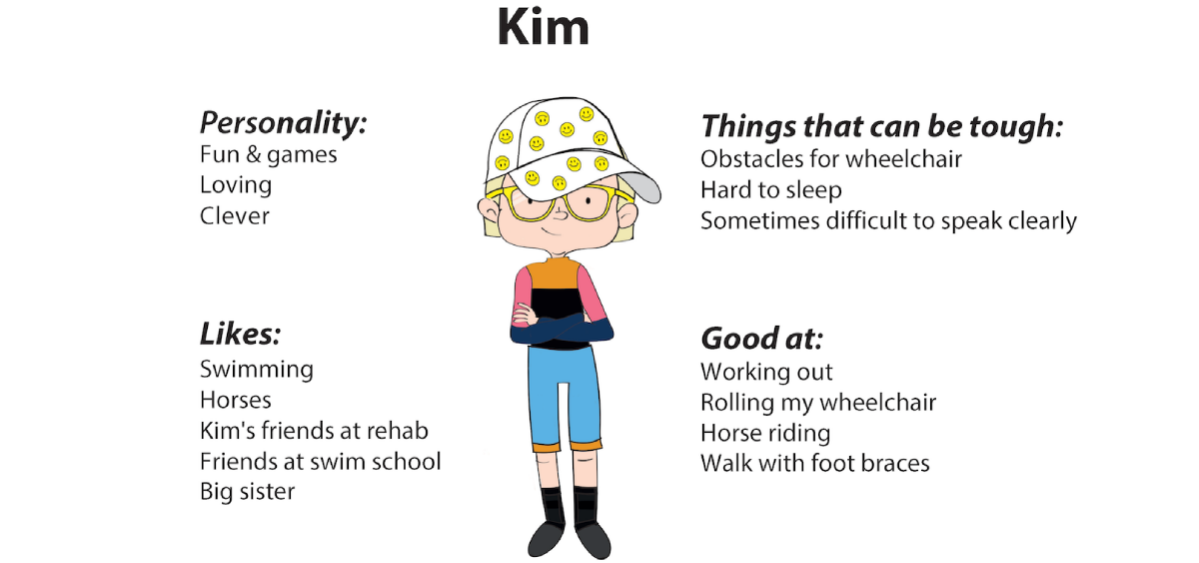
Example of a simplified persona used for the redemption scenarios in Workshop 2.

The purpose of Workshop 2 was to flesh out the personas by gaining insights on the participants’ problem-solving strategies in frustrating situations. Validation in these workshops (validation continued in Phase 3) entailed testing the credibility and usability of the personas. The participants co-created stories in the form of *redemption scenarios*, and we could see how the personas were perceived by the target group. The redemption scenarios were based on frustrations that had emerged in the characters from the previous workshops. Some contained more than one frustration. The first panel showed a situation, and the last panel showed that the situation was resolved and the persona was content. The children were asked to imagine how the story unfolds and connect the given beginning with the given end by filling out the empty panels in between. Six scenarios were designed, where 3 personas were represented in 2 scenarios each. Some related to rehabilitation situations but not all. It was considered more important to trigger strategic thinking in general, than in connection to certain situations. Other people in the scenarios were open for interpretation (who they were), and the scenarios used generic words like “exercise” rather than specifying “weight training,” “homework,” etc. This was to enable many children to be able to relate. Different interpretations of contexts might also generate a variety of strategies. The activity in this workshop was more demanding than previous workshops since the scenarios involved another person (the persona) and problem-solving. The workshops were thus individual to offer more support. Simplified versions of the personas were made in order to make them easy to understand (Figure 7). These were presented prior to the scenarios. The children chose which scenarios to work with. They either drew and wrote themselves, together with facilitators, or they had the facilitator to draw and write while co-creating the narrative. The facilitators discussed the persona’s character and interests, to trigger ideas in the participant. A little sketching by a facilitator could also stimulate the child’s creativity.

#### Workshop 2: Output and Analysis

**Figure 8:**
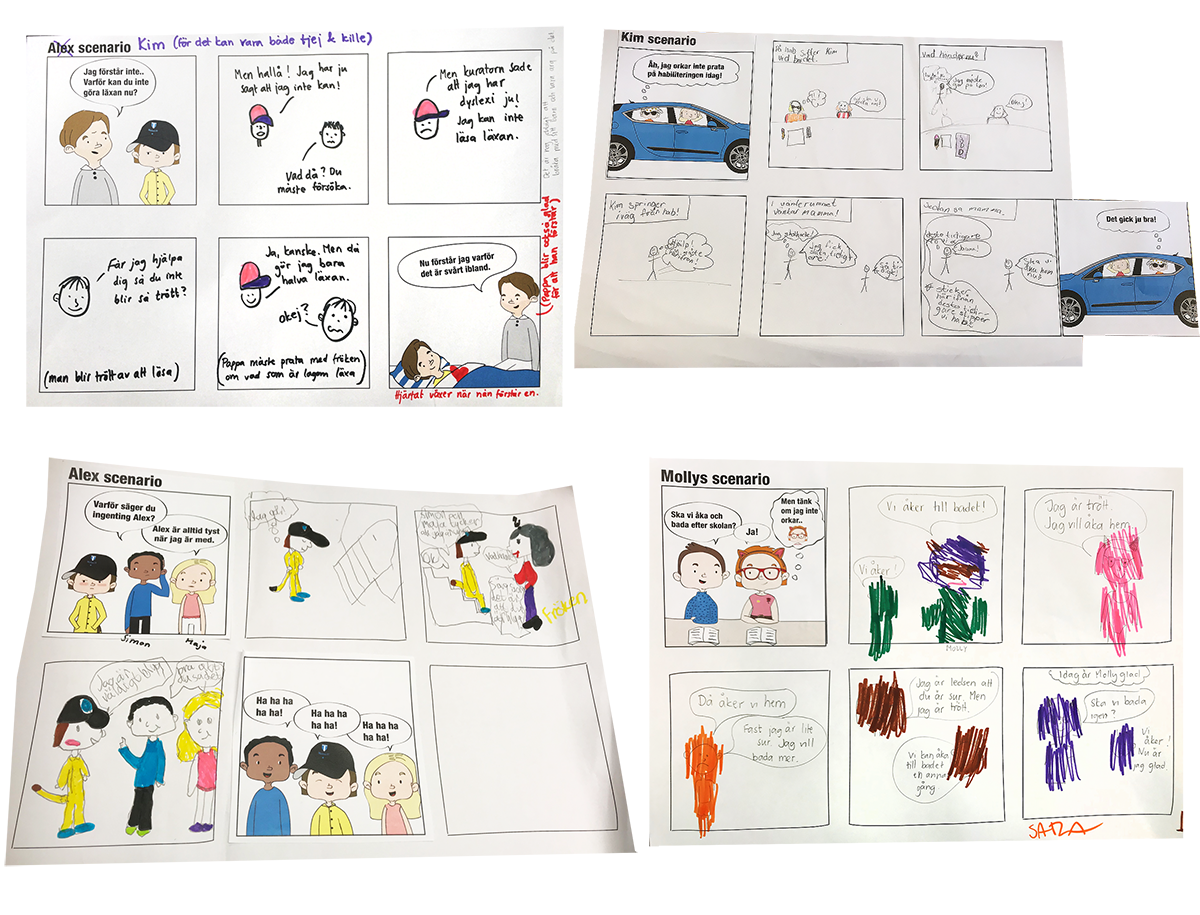
Examples of co-constructed redemption scenarios in Workshop 2.

**Figure 9:**
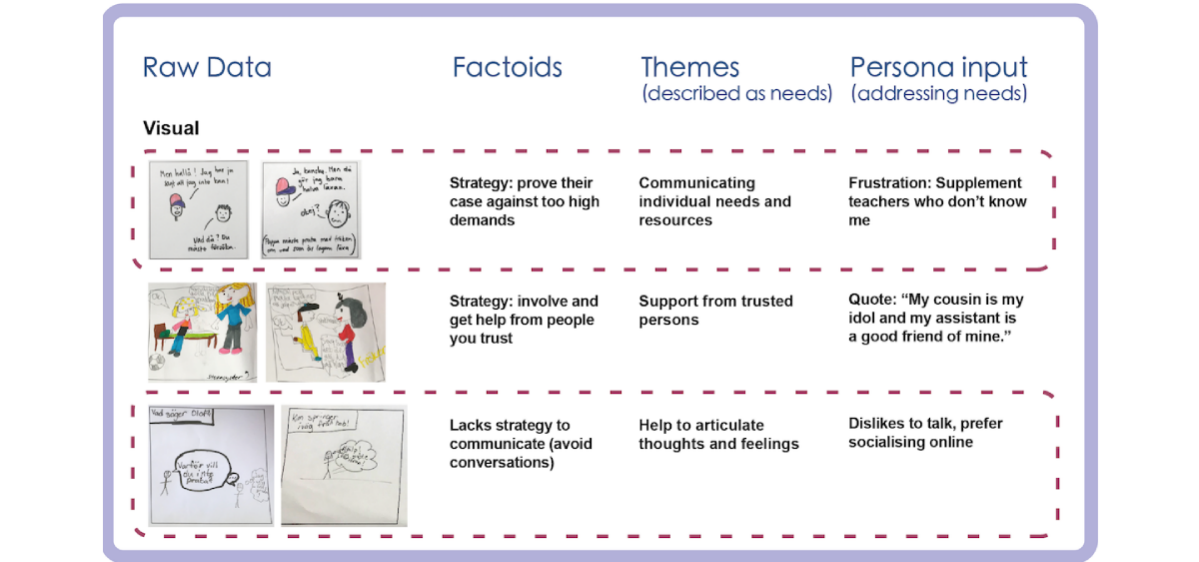
Examples from the data analysis of co-constructed redemption scenarios in Workshop 2.

The output of Workshop 2 was 8 redemption scenarios (Figure 8). The events in the children’s stories were abstracted into concepts of strategies (Figure 9). For example, people coming to rescue the persona were interpreted as *involve and getting help from people that you trust*. Simplified solutions (*lack of strategy*) were found too. While some children spent a lot of time coloring details, others quickly moved on to the next scenario. It was evident that children who had participated in Workshop 1 appreciated seeing details from their previous creations.

#### Modeling: Adjusting Personas

**Figure 10:**
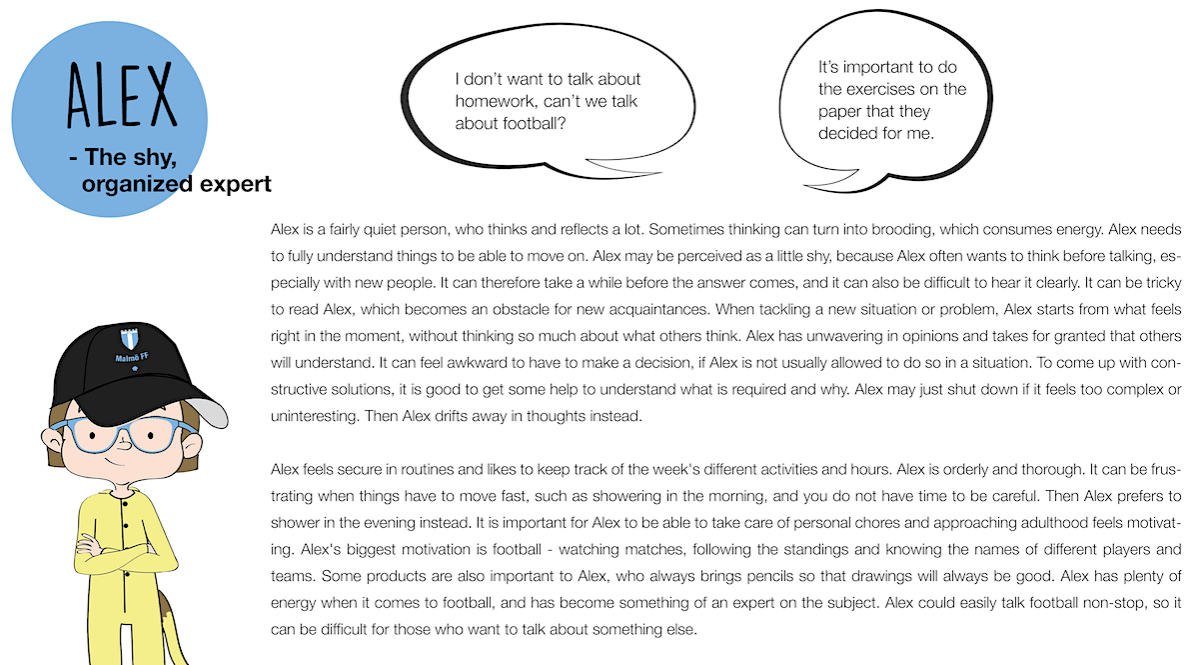
Persona additions after Workshop 2 (quotes, nickname, and story).

Based on the analysis of the redemption scenarios, the personas were slightly adjusted, and a story was created for each of them. In order to keep the personas open for different uses during the design process, the stories were not connected to a specific product or service. They were instead focused on the personas’ life contexts and served as a descriptive complement to the existing bullet points. The 3 versions (bullet point, story, and simplified persona) could be used for communication with different participants and stakeholders depending on the activity. The personas were also given descriptive nicknames to quickly convey their character (eg, *Alex – the shy, organized expert* and *Molly – a cheerful, short-tempered leader*). Each persona was completed with 2 quotes, such as “*It’s important to do the exercises on the paper, that they decided for me*” and “*I’m afraid I’ll say something stupid when I’m angry and lose friends*.” The personas were then ranked, which resulted in 1 primary persona and 2 secondary personas. This prioritization was based on needs and with the game for health in mind. The primary persona (*Alex*) was considered to have most to gain from the game in question. Accommodating *Alex’s* needs would probably also increase the appeal and usability for users in general. Figure 10 shows the persona additions after Workshop 2 (quotes, nickname, and story).

### Phase 3–Validation

#### Workshop 3: Question-led Brainstorming

Validation of the personas started in Workshop 2 as described above and continued in Workshop 3 through question-led brainstorming with children without disabilities. The primary persona was tested as a brainstorm tool in this workshop to confirm its credibility and usability. The purpose of the activity was to gain input and feedback on design sketches of the game for health. The persona *Alex* was presented in the simplified version (Figure 7) as a target user for the game. Sketches of the game were presented, and discussions around its theme, logic, and visuals were initiated. *Alex* was used to engage the children in inquiries such as “If Alex feels worried, how can Alex convey this within the game?” or “If Alex struggles with keeping focus, how should questions and rewards appear?” The workshop output consisted of drawings, notes, and audio recordings. The persona was effective in that the participants were able to discuss issues from *Alex’s* perspective as well as from their own, expressing, for example, “*Like, I’m thinking of Alex now, and football, referees, dunno… football players*” (when discussing supportive sidekicks in the game) and “*you shouldn’t focus on what you can’t do, but what you* can *do*.” Representation was perceived as important. The participants initiated discussions about how the sketches reflected norms related to appearance and the importance of representative illustrations to enable children to identify with figures in a game.

#### Validation Discussions

The personas were discussed with 1 young adult with a disability and 1 pediatric rehabilitation professional at the end of Phase 3. While the brainstorm workshops tested the primary persona, these discussions covered all 3 personas. This led to minor rephrasings such as replacing “wheelchair dependence” with “uses wheelchair” to connotate mobility rather than dependence. The overall perceptions of the 3 personas were summarized as follows:

I absolutely think they feel credible.

Young adult

[they] look good, many children/young people who are to be able to identify themselves and I think they’re comprehensive, which is positive!

Rehabilitation professional

The final personas are presented in Multimedia Appendix 3.

## Discussion

### Principal Findings

#### Persona Method Development

The method described involved development of existing persona generation and participatory design methods, adjusted to suit children with disabilities. The co-construction of characters and redemption scenarios offered a broad understanding of the participants’ life situation and approach, constituting a solid foundation for persona generation. Key factors were identified and addressed during the development. A substantial addition was the image bank, designed to motivate children and offer nonverbal construction of personas. The children were able to cope with a large number of images as long as they were arranged in a manageable way, and a clear activity overview was provided. Another component was the simplified persona versions, made to be comprehensible to child participants in participatory activities. The study was based on visual elements and proposes a thematic analysis for abstracting and translating image-based data into personas. We found that small groups and individual support helped children to stay focused, which is in line with other participatory research with children with disabilities [44]. An iterative process with numerous steps and co-creators implicated a gradual validation of the generated personas and their usability.

The norm-critical and social model approach also distinguishes this persona generation from established methods. For instance, disability was not explicit in our personas, who instead displayed assistive aids, context-bound barriers, or *frustrations*. This deviates from, for example, the report of Schulz and Fuglerud [53], who suggested that personas should display both the disability and its effects on life. However, the social model perspective resonated with the data where barriers were sometimes described as external factors, such as a lack of wheelchair access, or in quotes like “*my parents need help in understanding me*.” This contrasts pathogenic perspectives and norms regarding dysfunctions, their origins, and who has a problem. Similarly, since both gender and disability were regarded as partially socially constructed, some of the personas had no explicit gender. The fact that the target group was so diverse was an additional reason for keeping some characteristics open for interpretation and identification. Another deviant detail in our method was to use illustrations instead of photographs [56] to visually portray the personas. This was considered playful and enabled us to easily use elements from the children’s characters.

#### Finding the Right Level of Participation

If we acknowledge that people affected by design and technology should participate in its development, it is important to consider power imbalances and who has authority to select or neglect knowledge [12,68-70]. Although this study emphasizes increased participation for children with disabilities, it might not satisfy demands within participatory research for participant involvement through *all* stages, including the analysis [34,68]. Skills and capabilities are often carefully considered for this group to determine suitable participation levels for each participant [11,43,44]. Caution in terms of extensive activities for vulnerable participants has also been discussed in this paper, as children were not involved in all the analysis and validation steps. Instead, numerous participants and iterations of activities meant a continuous adjustment and a gradual validation of the analytical output. Furthermore, since disability is just one of many variables for our target group, they share many preferences with children without disabilities. Users outside of the target group were thus considered as valid representatives for some of the activities [47,49].

#### Recruitment and Representation

The difficulty in accessing and recruiting child patients and participants with disabilities has been recognized in previous research [9,46,49]. Both parental consent and other forms of engagement were necessary in this study, such as providing transportation to an activity. This can constitute a risk that the participants only represent groups where parents have an interest in increased child participation as well as have the resources to allow them to take part. Children’s voices in research are normally conditioned by parental consent, which poses a dilemma since it narrows the spread of perspectives being heard [71]. One way to reach participants with diverse backgrounds in this study was to conduct some workshops at schools. It has been suggested that being situated within a school environment could hinder full participation due to lingering power hierarchies in the buildings [47] and that children might only do the minimum required [43]. Although this was not perceived to be a problem for the activity, it is difficult to rule out that motivation was lower in the school workshops.

#### Norm-Creative Tradeoffs

Bearing in mind that materials used in design processes inevitably shape their result [72], we paid attention to how the imagery portrayed family setup, gender, ethnicity, and functionality to avoid producing norm-affirmative output or stereotypical narratives. It was sometimes a question of balancing representation on the one hand, and comprehensibility or manageability on the other. We thus had to use some archetypes and a limited variety in the image bank. This limitation could be partly addressed by being attentive to the participants’ preferences and encouraging them to add to the material. However, should this method be transferred to more diverse contexts, we suggest a more varied representation, for example, in the skin tone of characters. The designed material is thus not fixed but highly malleable to enable flexible applications.

### Implications

*Norm-criticism* raises awareness of norms that exclude or discriminate, while *norm-creativity* is a combined approach requiring both norm-critical awareness and design thinking, with the aim to move beyond or counteract norms [39]. As norm-criticism influenced this study’s creative process, the resulting method has potential to shape norm-creative solutions in projects using it. However, norm-critical awareness must accompany its use as the method does not replace critical thinking in the researcher. As highlighted by Pruitt and Grudin [56], personas must be used ethically, as with all scientific methods. Personas are tools for both design thinking and norm challenging, which may occur during persona construction or in the use of personas. Discussions triggered by a norm-creative persona generation method can also generate reflexivity within research [71,73]. The described method enables increased influence for children with disabilities in research and design processes. This might in the long run also influence norms of decision-making within such contexts.

The method was developed to suit children aged 6 to 17 years with disabilities, but while the method might be transferable to similar groups, age might not solely dictate who finds it beneficial. Personality, physical abilities, or cognitive development could be equally important [12]. The facilitators’ resources and communicative skills could also affect the possibilities for participation. While the results were influenced by a game for health context, the method can be adjusted to suit other design contexts too. There are unlimited possibilities in terms of materials that can help include participants. One limitation of this study was that no alternative materials or settings were compared. Future research could thus involve other contexts or user groups, as well as comparisons and usage of differently generated personas.

### Conclusions

This paper describes the development of a participatory persona generation method aimed to suit children with disabilities. The method strives to enable and capture the perspectives of this group by using iterative workshops and flexible materials. The results provide guiding examples for image-based workshops and analysis. Combined with norm-critical awareness, the method has potential to influence design projects in the direction of increased representation, norm-creativity, and inclusiveness. The method was developed within a games for health case, through which it was contextualized and validated. It may also be suited for, or adjusted to, similar contexts or user groups. This could be subject to further research.

## Appendix

Multimedia Appendix 1 Proxy/skeleton personas.

Multimedia Appendix 2 Project overview and workshop agenda.

Multimedia Appendix 3 Final personas.
