# Supplementary material for: A Norm-Creative Method for Co-constructing Personas With Children With Disabilities: Multiphase Design Study
Source: J Particip Med. 2022 Jan 6;14(1):e29743. doi: 10.2196/29743 (PMC8778551; doi:10.2196/29743)
Supplement: Multimedia Appendix 1 [file jopm_v14i1e29743_app1.pdf]

LISA

I AM GOOD AT
- Speak my mind and represent others at class meetings
- Draw and paint

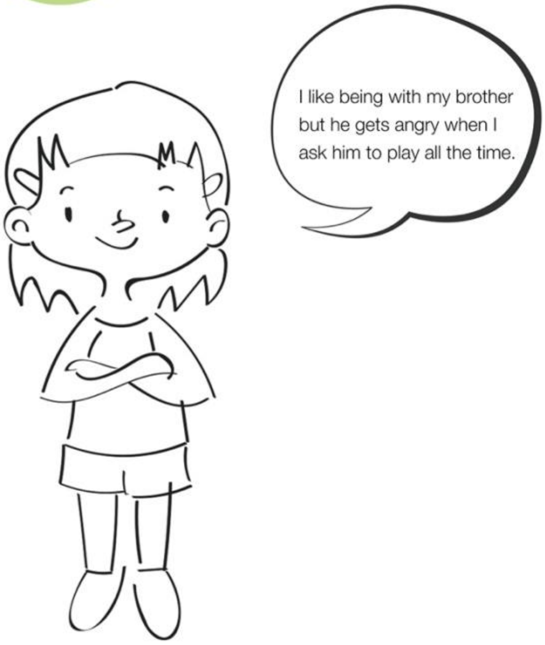

PERSONALITY
- Intense
- Jokey
- Keep the group together

FAMILY
- Mum, dad
- Stays most at mum
- Little sister, brother
- Far from dad to rehab

FRUSTRATIONS
- When hands cramp
- When text and numbers blur
- Supply teachers who don't know me
- Getting tired all the time

MOTIVATIONS
- When people laugh at my jokes
- To see that I grow when I eat well

GOAL
- Have my own horse

PRODUCTS
- Want a sturdy bike
- Good at tech stuff
- Not too childish toys
- Grip aid for phone

I LIKE
- Draw, colors and forms
- Green and purple
- Horses

I DON'T LIKE
- Noisy class room, I can't think
- When dad gets angry
- Big spaces, crowds

DAVID

I AM GOOD AT
- Calculate and remember numbers
- Memorize cities and countries
- Build Lego figures

GOAL
- To be a train maker
- To manage on my own

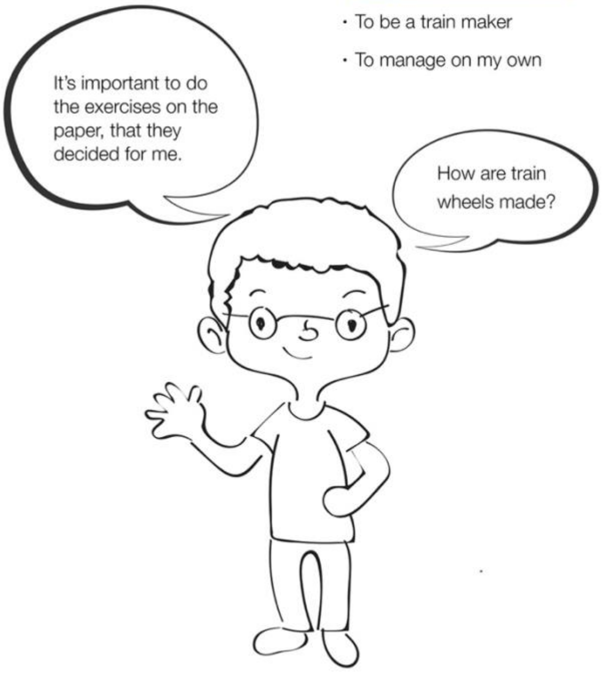

PERSONALITY
- Ask questions and look up facts about my interests
- Careful
- Want to manage things, refrain from doing things if risking failure
- Low self confidence
- Like hanging out with grownups, like cousin and the school caretaker

FAMILY
- Grandma and grandpa
- Mum, dad
- Big brother

FRUSTRATIONS
- When I have to sit long and do things I'm not interested in, then I shut down.
- When teachers won't let me use my screen reader on tests.
- Hard to see who is in my team at PE

MOTIVATIONS
- To follow things being built
- To reach goals I have set up
- Build a Millenium Falcon

PRODUCTS
- Building blocks
- Screen reader
- Costumized keyboard

I LIKE
- Watches
- Build with Lego
- Stay at Grannie
- Games
- Meatballs and pasta
- To know what the plan is
- Music

I DON'T LIKE
- Large crowds
- Talk to people I don't know
- Whan plans change

This is a Multimedia Appendix to a full manuscript published in the J Med Internet Res. For full copyright and citation information see doi: 10.2196/29743

OLLE

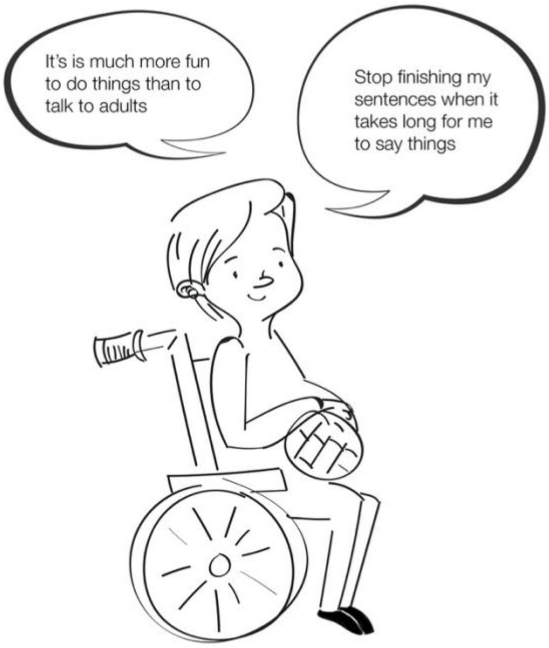

I AM GOOD AT

- Football, score, and goal keeper sometimes
- Read and write

PERSONALITY

- Active
- Like challenges

FAMILY

- Mum, dad
- Cousin (idol)

FRUSTRATIONS

- When schoolyard is not adjusted to my wheelchair
- When I forget things
- To not be able to to anything myself
- When others don't understand me

MOTIVATIONS

- When I can join my friends at PE
- When my parents think I'm doing well
- Manage to read Lord of the Rings

GOAL

- To score goals

PRODUCTS

- Wheelchair (want new one, bad design for steering, afraid to fall)
- New phone, proud of

I LIKE

- Football (watch, and play)
- Phone
- Be with cousin and friends
- Write and answer on social media
- Lots of color
- Fantasy books and films
- Play mobile games

I DON'T LIKE

- Iphone
- Work out (unless it's football)

This is a Multimedia Appendix to a full manuscript published in the J Med Internet Res. For full copyright and citation information see doi: 10.2196/29743
