# Supplementary figures and images for: A Norm-Creative Method for Co-constructing Personas With Children With Disabilities: Multiphase Design Study
Source: J Particip Med. 2022 Jan 6;14(1):e29743. doi: 10.2196/29743 (PMC8778551; doi:10.2196/29743)

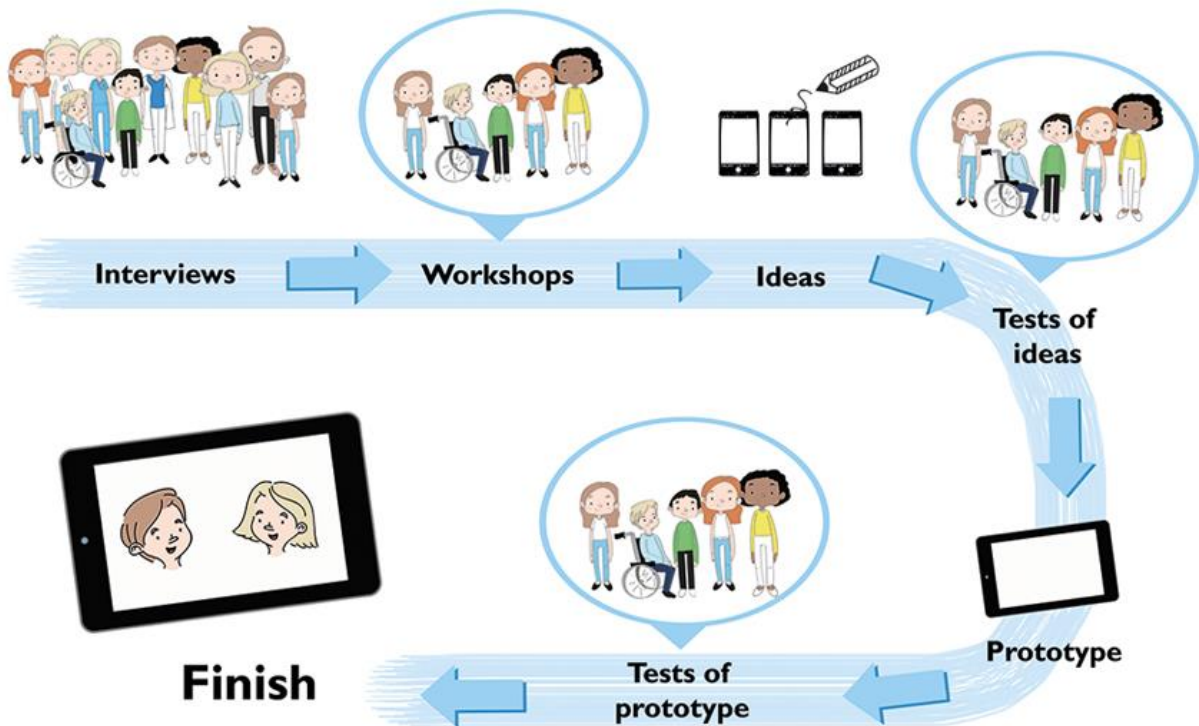

# 1 WHO ARE WE?

# 2 WHY ARE WE HERE?

# 3 ACTIVITY

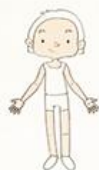

- CREATE CHARACTERS
- WHAT DO CHARACTERS DO DURING A DAY?

# 4 WRAP UP

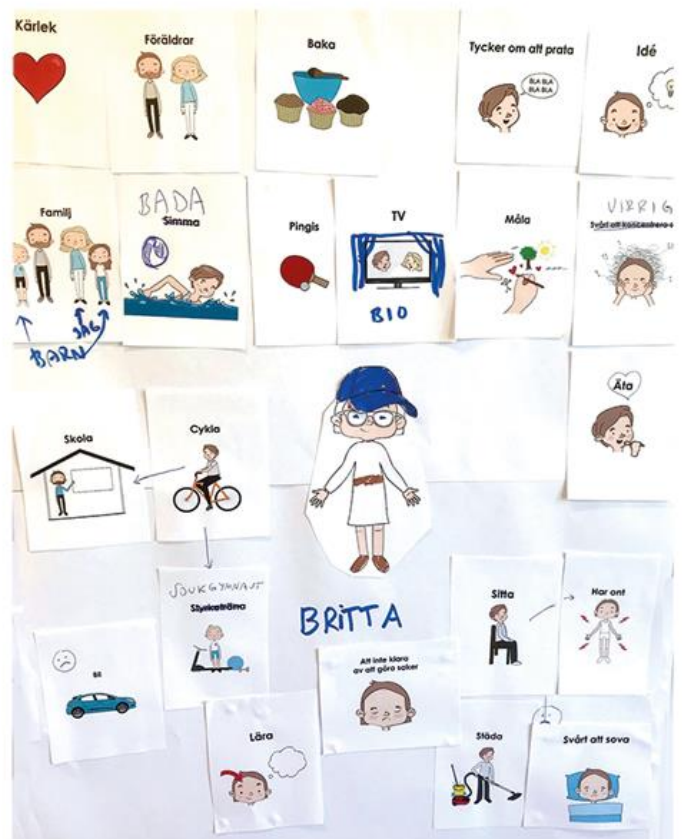

Supplement: Multimedia Appendix 2 [file jopm_v14i1e29743_app2.pdf]
