# Supplementary material for: A Norm-Creative Method for Co-constructing Personas With Children With Disabilities: Multiphase Design Study
Source: J Particip Med. 2022 Jan 6;14(1):e29743. doi: 10.2196/29743 (PMC8778551; doi:10.2196/29743)
Supplement: Multimedia Appendix 3 [file jopm_v14i1e29743_app3.pdf]

# Final personas

## ALEX
**The shy, organized expert**

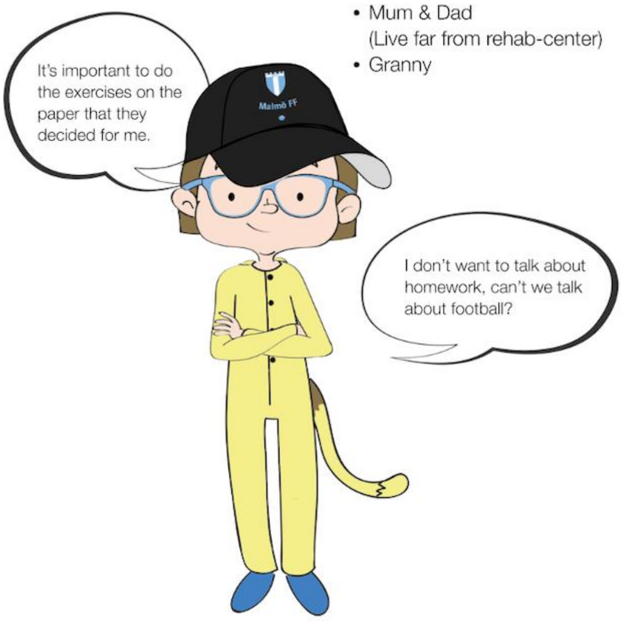


### I AM GOOD AT
- TV games (like minecraft)
- Football facts

### FAMILY
- Mum & Dad (Live far from rehab-center)
- Granny

### PERSONALITY
- Shy (low self confidence)
- Think a lot, dwell on things
- One thing at a time
- Careful
- Curious, find things out
- Enjoy adult company (Assistant, neighbour...)

### FRUSTRATIONS
- Tired after school
- Concentration disappears when things get boring
- Others don't understand my feelings
- When teathers don't let me use screen-reader on tests
- Hard to shift focus
- Difficult to explain and articulate so that others understand

### GOALS
- Take care of myself
- Set my own goals and reach them

### MOTIVATION
- Football

### PRODUCTS
- My pens
- Screen reader

### I LIKE
- Fotboll
- TV series
- Sleep
- Hangout with my neighbour
- My new glasses

### I DON'T LIKE
- Talking to people I don't know

## ALEX
**- The shy, organized expert**

I don't want to talk about homework, can't we talk about football?

It's important to do the exercises on the paper that they decided for me.

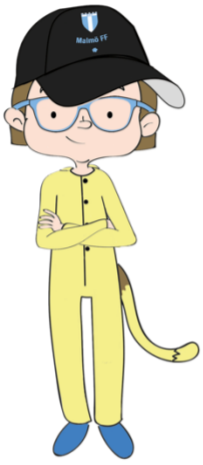


Alex is a fairly quiet person, who thinks and reflects a lot. Sometimes thinking can turn into brooding, which consumes energy. Alex needs to fully understand things to be able to move on. Alex may be perceived as a little shy, because Alex often wants to think before talking, especially with new people. It can therefore take a while before the answer comes, and it can also be difficult to hear it clearly. It can be tricky to read Alex, which becomes an obstacle for new acquaintances. When tackling a new situation or problem, Alex starts from what feels right in the moment, without thinking so much about what others think. Alex has unwavering in opinions and takes for granted that others will understand. It can feel awkward to have to make a decision, if Alex is not usually allowed to do so in a situation. To come up with constructive solutions, it is good to get some help to understand what is required and why. Alex may just shut down if it feels too complex or uninteresting. Then Alex drifts away in thoughts instead.

Alex feels secure in routines and likes to keep track of the week's different activities and hours. Alex is orderly and thorough. It can be frustrating when things have to move fast, such as showering in the morning, and you do not have time to be careful. Then Alex prefers to shower in the evening instead. It is important for Alex to be able to take care of personal chores and approaching adulthood feels motivating. Alex's biggest motivation is football - watching matches, following the standings and knowing the names of different players and teams. Some products are also important to Alex, who always brings pencils so that drawings will always be good. Alex has plenty of energy when it comes to football, and has become something of an expert on the subject. Alex could easily talk football non-stop, so it can be difficult for those who want to talk about something else.

---

Alex's traits come from meetings with, and material created by, young people with autism and interviews with their parents. Difficulties with speaking and reading texts apply to several children who have been involved in this project. That concentration runs out quickly is common for most of these young people. But for Alex it depends a lot on whether it is interesting or not. If it is of interest, focus can last a very long time. Not understanding one's own feelings is an obstacle if one is expected to explain oneself to others. For Alex this difficulty is related to both closedness, shyness and other feelings.

This is a Multimedia Appendix to a full manuscript published in the J Med Internet Res. For full copyright and citation information see doi: 10.2196/29743

# MOLLY

**- a cheerful, short-tempered leader**

I'm afraid I'll say something stupid when I'm angry, and lose friends

I'm good at speaking up, so I am a class representative at school

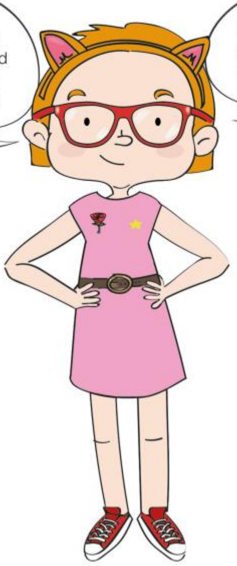

## I AM GOOD AT

- Dance
- Baking cakes
- Speak my mind
- Draw and paint

## FAMILY

- Mum (mostly living with)
- Dad (lives far from us)
- Little sister

## PERSONALITY

- Talkative, intense
- Jokey
- Get angry sometimes
- Keep the group together

## FRUSTRATIONS

- When hands cramp
- Tired = loose my temper
- Loose focus quickly
- Hard to read, blurry
- Supply teachers who don't know me
- Often tired
- Busy, noisy classroom

## GOAL

- My own horse

## MOTIVATIONS

- I can read books if they are really exciting
- When people laugh at my jokes
- To see that I grow when I eat well

## PRODUCTS

- Grip aid for my phone

## I LIKE

- Cats
- Friends
- Cafes
- Horses
- Music

## I DON'T LIKE

- Crowds
- Large spaces
- When my parents are angry

# MOLLY

**- A cheerful, short-tempered leader**

I'm afraid I'll say something stupid when I'm angry, and lose friends

I'm good at speaking up, so I am a class representative at school.

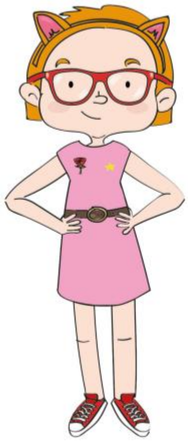

Molly is often the one who talks, jokes with everyone and is a bit loud. She is a leader who wants everyone to be in a good mood. Molly is good at speaking her mind and standing up for those who do not dare to. She is also creative and likes to bake, draw and paint. Molly is also very fond of animals. She goes with the flow, and lives in the moment.

Her various diagnoses often make her tired. Molly would need to manage her energy better, because when it runs out or her hands cramp, Molly loses her temper and can get really angry. With a dad who (also) has ADD, it can be noisy at the weekends she stays with him. There is also a concern in Molly that she will hurt those she likes when she is angry. School is not Molly's favorite place, as there are large and often noisy classrooms, texts are difficult and tiring to read, and supply teachers do not always know Molly's needs.

Something that motivates her is exciting books and attention from friends. She is a bit careless with her food, but works on eating better. She gets extra motivation for it when she sees that it gives results.

---

Molly's persona is in her teens, and incorporates traits from young people who are a little tired of school, intense and in the center of things. Molly has difficulties associated with dyslexia, ADD, and muscle disease. In the target group, it is common for several diagnoses to exist within the same family, which can affect the child's ability to manage his or her needs.

This is a Multimedia Appendix to a full manuscript published in the J Med Internet Res. For full copyright and citation information see doi: 10.2196/29743

# KIM
**- Determined and playful**

Fun and games is much better than talking to grownups!

Stop finishing my sentences, it sometimes takes long for me to say things

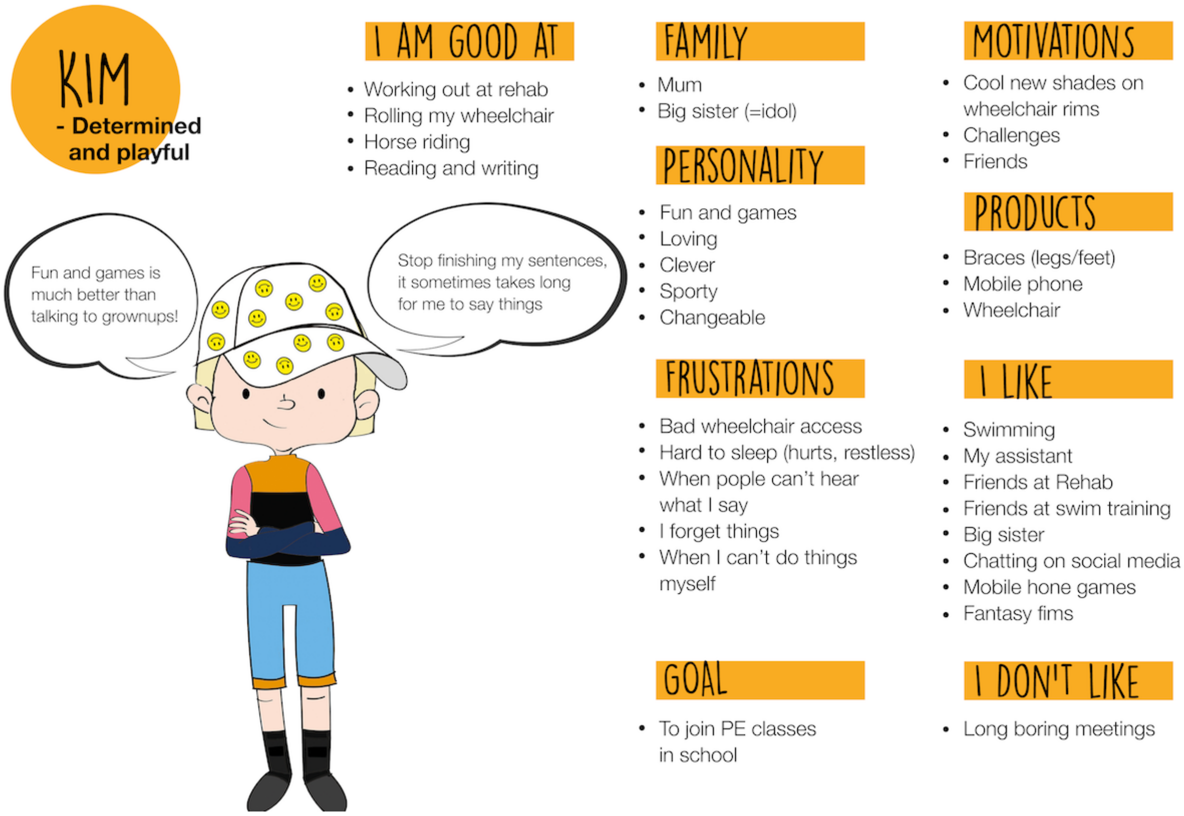

## I AM GOOD AT
- Working out at rehab
- Rolling my wheelchair
- Horse riding
- Reading and writing

## FAMILY
- Mum
- Big sister (=idol)

## PERSONALITY
- Fun and games
- Loving
- Clever
- Sporty
- Changeable

## FRUSTRATIONS
- Bad wheelchair access
- Hard to sleep (hurts, restless)
- When pople can't hear what I say
- I forget things
- When I can't do things myself

## GOAL
- To join PE classes in school

## MOTIVATIONS
- Cool new shades on wheelchair rims
- Challenges
- Friends

## PRODUCTS
- Braces (legs/feet)
- Mobile phone
- Wheelchair

## I LIKE
- Swimming
- My assistant
- Friends at Rehab
- Friends at swim training
- Big sister
- Chatting on social media
- Mobile hone games
- Fantasy fims

## I DON'T LIKE
- Long boring meetings

# KIM
**- Determined and playful**

Fun and games is much better than talking to grownups!

Stop finishing my sentences, it takes long for me to say things sometimes!

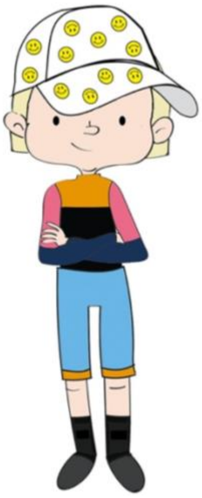

Kim is a loving kid, full of energy. Kim is street smart and likes challenges. There is need and interest in Kim to express his/her personality, for example with clothes and accessories. Confirmation from others is also important, for example when Kim has been good at working out, or done well at school. Kim is motivated by goals. One is to be able to tie the sneaker shoelaces to be able to participate in sports with the other children. Kim is also social and communicates easily, both on social media and in other environments - school, rehab, swim school, roller school and more. However, it can be difficult to speak clearly, and Kim can get sad if others do not understand. Digital forums are better in that sense. Both laughter and crying come easily, and the mood changes quickly. Kim has great support in a big sister, who is also Kim's idol and best friend. It is no problem for Kim to take responsibility or make decisions. S/he has many good ideas and solutions and is not afraid to try to influence the outside world. Kim is active and likes to play games on TV/mobile/tablet. Rehab meetings are difficult though, they are too long and boring. Kim has leg pain sometimes, especially at night which causes sleeping difficulties and fatigue. It can be frustrating not to be able to do something you want. Sometimes Kim gets forgetful too, which irritates Kim.

---

Kim's persona is based on personalities and functional variations in several children, such as CP which can lead to pain, speech difficulties and wheelchair use. Mood swings occurred during the project's workshops, and regardless of why they will affect the child and the social environment. Having many activities outside of school is not unusual for our target group, something that can both give and take energy. Kim has gotten an attitude from some of these children – believing that anything is possible as long as you are a little inventive and get help from people.

This is a Multimedia Appendix to a full manuscript published in the J Med Internet Res. For full copyright and citation information see doi: 10.2196/29743
